# Identification of novel and robust internal control genes from *Volvariella volvacea* that are suitable for RT-qPCR in filamentous fungi

**DOI:** 10.1038/srep29236

**Published:** 2016-07-12

**Authors:** Yongxin Tao, Arend Frans van Peer, Qianhui Huang, Yanping Shao, Lei Zhang, Bin Xie, Yuji Jiang, Jian Zhu, Baogui Xie

**Affiliations:** 1 College of Horticulture, Fujian Agriculture and Forestry University, Fuzhou, 350002, Fujian, China; 2 Mycological Research Center, College of Life Sciences, Fujian Agriculture and Forestry University, Fuzhou, 350002, Fujian, China; 3 College of Food Science, Fujian Agriculture and Forestry University, Fuzhou, 350002, Fujian, China

## Abstract

The selection of appropriate internal control genes (ICGs) is a crucial step in the normalization of real-time quantitative PCR (RT-qPCR) data. Housekeeping genes are habitually selected for this purpose, despite accumulating evidence on their instability. We screened for novel, robust ICGs in the mushroom forming fungus *Volvariella volvacea*. Nine commonly used and five newly selected ICGs were evaluated for expression stability using RT-qPCR data in eight different stages of the life cycle of *V. volvacea*. Three different algorithms consistently determined that three novel ICGs (*SPRYp*, *Ras* and *Vps26*) exhibited the highest expression stability in *V. volvacea*. Subsequent analysis of ICGs in twenty-four expression profiles from nine filamentous fungi revealed that *Ras* was the most stable ICG amongst the Basidiomycetous samples, followed by *SPRYp*, *Vps26* and *ACTB*. *Vps26* was expressed most stably within the analyzed data of Ascomycetes, followed by *HH3* and *β-TUB*. No ICG was universally stable for all fungal species, or for all experimental conditions within a species. Ultimately, the choice of an ICG will depend on a specific set of experiments. This study provides novel, robust ICGs for Basidiomycetes and Ascomycetes. Together with the presented guiding principles, this enables the efficient selection of suitable ICGs for RT-qPCR.

Real-time quantitative PCR (RT-qPCR) has emerged as a powerful and popular tool for rapid and accurate assessment of changes in gene expression^1^,^2^. The reliability of gene expression that is determined by RT-qPCR is strongly affected by technical factors like quality of RNA, efficiency of cDNA synthesis, primer performance, and normalization^3^,^4^,^5^. For proper normalization of target gene expression, especially in samples with fluctuating expression levels, the selection of a stable internal control gene (ICG) is extremely important^6^,^7^. Traditionally, housekeeping genes are selected for this purpose. Accumulating evidence indicates that most of these housekeeping genes, while stable under certain conditions^8^,^9^, exhibit irregular or even largely differential expression under other conditions^10^,^11^,^12^,^13^,^14^. Equally important are the absolute expression levels of ICGs, which are often neglected. Large differences in absolute expression between an ICG and a target gene will exaggerate or obscure the actual differences in target gene expression levels between samples^15^. Large differences in absolute expression will also force either the ICG, or the target gene, into an unreliable Ct value range.

Filamentous fungi are important organisms that are frequently studied by RT-qPCR, yet identification of suitable ICGs has received little attention. Existing reports on fungal ICGs mainly focus on relative expression stability^16^,^17^,^18^, and ignore absolute expression levels. Moreover, these studies mostly involve traditional housekeeping genes in Ascomycetes^10^,^14^,^16^,^17^,^18^,^19^ and except for *Phakopsora pachyrhizi*^20^ and *Pleurotus ostreatus*^21^; little information is available on ICGs in Basidomycetes. The available information is furthermore of limited value for new species, as most ICG studies have been restricted to a single species under specific experimental conditions^10^,^19^. The identification of novel and robust ICGs that are suitable for multiple filamentous fungi would therefore be an important improvement. In this study we identified known and novel ICGs in the mushroom forming fungus *Volvariella volvacea*, and assessed their suitability in various filamentous fungi. *V. volvacea*, also known as the straw mushroom or Chinese mushroom, is cultivated on a large scale for its unique flavor and high nutritious value^22^. Its short life cycle (about twenty days under laboratory conditions) and the characteristic five-step development of its fruiting bodies (primordia, button stage, egg stage, elongation stage and maturation stage, according to Chang^23^), make it a suitable model for molecular genetic studies on fruiting body development. The different stages and tissues of this mushroom are associated with large variations in gene expression. The already available stage specific and tissue specific digital gene expression profiles (DGEs) of this mushroom therefore provided an excellent background for the analysis of ICGs. Using the DGEs, ICGs were selected regarding their absolute expression levels and non-differential expression between the various developmental stages. Subsequently, the stability of the selected ICGs was determined based on analysis of RT-qPCR data with three different statistical algorithms; geNorm^24^, NormFinder^25^ and BestKeeper^26^. Finally, the selected *V. volvacea* ICGs were examined for their stability in other filamentous fungi.

## Results

### Selection of housekeeping genes and novel ICGs

Nine housekeeping genes that are traditionally used as ICGs were identified in the genome of *V. volvacea* and confirmed by BLAST in NCBI: 18S rRNA (*18S*), β-actin (*ACTB*), cyclophilin (*CYP*), glyceraldehyde-3-phosphate dehydrogenase (*GAPDH*), histone H3 (*HH3*), tubulin (*α-TUB*, *β-TUB1*, *β-TUB2*) and ubiquitin (*UBQ*). All of these genes (*18S* was not represented in the DGEs) exhibited differential expression between one or more stages (Supplementary Table S1).

For the five novel ICGs, we screened the expression profiles of eight different developmental stages in *V. volvacea*. The represented stages included the mycelium of the two parental homokaryons, mycelium of the heterokaryon, and differentiated tissue of the five characteristic mushroom stages: primordia, button, egg, elongation and maturation. In total, 9,338 genes of the 11,534 predicted genes in the *V. volvacea* genome were expressed in at least one stage. Of the 9,338 genes, 2,681 were non-differentially expressed between any of the eight stages. Sixty-five of the non-differentially expressed genes showed a minimum expression of ten tag counts or more in all expression profiles (genes with fewer than ten tag counts were considered to be expressed too low to be good ICG). Twenty-four genes with a predicted function (forty-one of the sixty-five were either predicted or hypothetical genes) were ranked according to their TPM (transcripts per million clean tags) values and their similarity to genes in other fungi (i.e. having the lowest *E* values). The top five of these genes, *L-asp*, *Ras*, *SPRYp*, *Vps26* and *MSF1*, were selected for further analysis (Table 1). The average TPM values of the five genes represented absolute expression levels of varying orders of magnitude (Table 1). Moreover, all five newly selected ICGs contained conserved domains and were represented in plants (including *Arabidopsis thaliana* and *Oryza sativa*), fungi (yeasts and filamentous fungi) and mammals (including humans). In addition, each was predicted to be involved in basic cellular processes. *L-asp* encodes L-asparaginase, a key enzyme in amino acid metabolism and the formation of aspartic acid^27^. *Ras* is an important member of the small GTPase family that functions as a signaling hub for GDP/GTP molecular switches in cellular signal transduction^28^,^29^. *SPRYp* and *Vps26* are involved in endosome-vacuole trafficking and vacuolar protein sorting^30^,^31^,^32^,^33^. *MSF1* is involved in intra-mitochondrial protein sorting and phosphatidylethanolamine (PE) metabolism^34^. Their moderate expression, high conservation, and basic cellular function suggested that the five genes could be interesting ICGs for a range of species.

### RT-qPCR analysis of ICGs in eight developmental stages of *V. volvacea*

**Figure 1:**
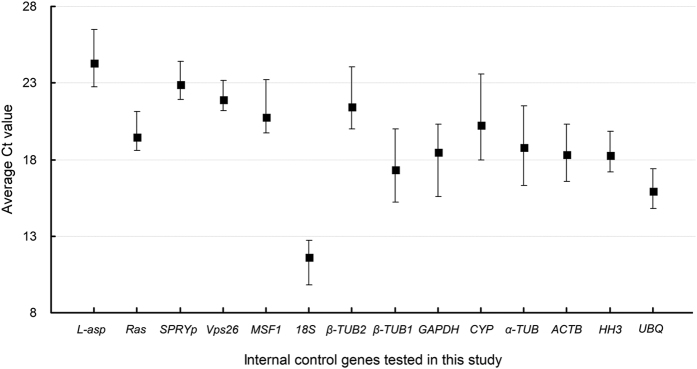
Variation in RT-qPCR values of fourteen ICGs in eight samples of *V. volvacea*. Mean Ct values of each ICG in eight samples (three mycelium samples: strain PYd15, strain PYd21, strain H1521; and five developmental stages of fruiting bodies of the *V. volvacea* heterokaryon H1521: primordia, button stage, egg stage, elongation stage and maturation stage) are shown on the y-axis. Error bars represent the maximum and minimum Ct values, respectively. The fourteen candidate ICGs are shown on the x-axis.

The precise expression levels of the fourteen selected ICGs were evaluated based on RT-qPCR. The amplification efficiencies (E) of the fourteen genes were calculated from the slopes of their standard curves and varied from 87.3% for *18S* to 112.6% for *Ras* (Table 2). All fourteen correlation coefficients (R^2^) were greater than 0.99. This indicated a reliable linear relationship between the respective Ct values and the log values of the initial gene copy numbers. The average Ct values of the ICGs (Fig. 1), calculated over the eight different stages of *V. volvacea*, showed that *18S* was considerably higher expressed than the other ICGs (low Ct values), while *L-asp* was expressed lowest (high Ct values) in all but one sample (the elongation stage, Supplementary Fig. S1). Most genes showed trends of higher or lower expression relative to the other ICGs (Fig. 1). The relative variation of the Ct values (the maximum Ct value minus the minimum Ct value) for each of the ICGs over the eight samples showed the greatest variation for *β-TUB1*, *GAPDH*, *CYP* and *α-TUB*, and the smallest variation for three of the five novel ICGs (*Ras*, *SPRYp* and *Vps26*) in *V. volvacea* (Fig. 1).

### Evaluation of expression stability of fourteen ICGs in *V. volvacea*

The stability of the fourteen ICGs was evaluated based on RT-qPCR Ct values using three statistical algorithms: geNorm, NormFinder and BestKeeper. Four sample sets (A, B, C and D) comprising varying combinations of the eight *V. volvacea* stages (Supplementary Table S2) were used for geNorm and NormFinder analysis to evaluate the effect of different sample compositions in experiments. For BestKeeper analysis, the seven most stable genes were compared only, to comply with the limitations of the program.

#### GeNorm analysis of ICGs

**Figure 2:**
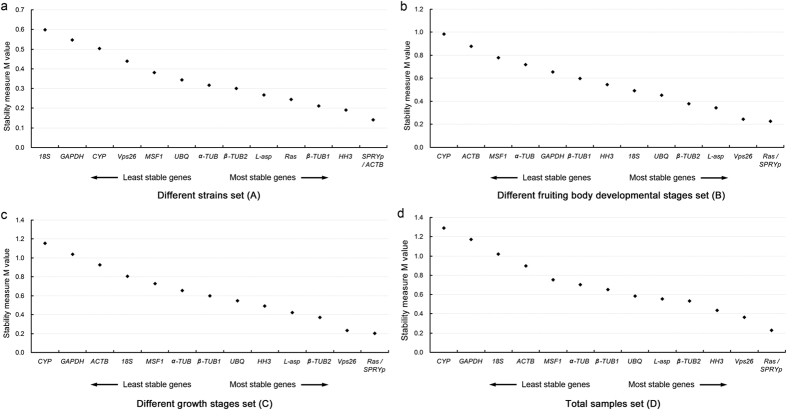
Expression stability ranking of fourteen candidate ICGs using geNorm. Stability measure M values, calculated by geNorm, are shown on the y-axis. The lowest M values represent the most stable expression; (**a**–**d**) represent four different combinations of sample sets (see Supplementary Table S2).

GeNorm calculates the gene expression stability measure M of an ICG based on the average pairwise variation between all studied ICGs. Lower M values (i.e. less average variation) indicate higher expression stability^24^. The assigned M values of the fourteen ICGs varied most between set A and sets B to D (Fig. 2). For mycelium of different strains (set A), *SPRYp* and *ACTB* were indicated as the most stable genes, while *18S* and *GAPDH* were indicated as least stable (Fig. 2a). Within the developmental stages of the fruiting body (set B), *Ras* and *SPRYp* were most stable, while *CYP* and *ACTB* were clearly less consistently expressed (Fig. 2b). For sets C (mycelium plus mushroom developmental stages) and D (the total samples set), *Ras* and *SPRYp* were also indicated as the most stable genes, and *CYP* and *GAPDH* were indicated as least stable (Fig. 2c,d). *SPRYp* was thus the most stably expressed gene in all test samples in *V. volvacea* according to geNorm. While *ACTB* was indicated as one of the most stably expressed genes in set A, it was far less stable in set B (second last), set C (third last) and set D (fourth last), indicating that specific combinations of samples can have a substantial influence on the suitability of an ICG. Notably, the commonly used ICGs *CYP*, *18S* and *GAPDH* were indicated as the least stable genes in the four sample sets of *V. volvacea*.

GeNorm also calculated a normalization factor to determine the optimal number of ICGs for accurate normalization, based on pairwise variations (V_n/n + 1_) between each combination of sequential normalization factors. The highest pairwise variations for any given ICG pair were 0.072 for set A, 0.112 for set B, 0.124 for set C, and 0.148 for set D. According to the default cut-off value of 0.15 for pairwise variations^24^ (the inclusion of an additional reference gene would not be required if pairwise variation is smaller than 0.15), two genes would suffice for accurate normalization in all four sample sets of *V. volvacea*. The optimal combination of ICGs for the different strains (set A) was *SPRYp* with *ACTB*, while for the three remaining sets (B, C and D) the combination of *SPRYp* and *Ras* was indicated to be optimal.

#### NormFinder analysis of ICGs

NormFinder, which uses a model-based approach to rank ICGs based on inter- and intra-group expression variations^25^, invariably predicted *SPRYp* to be the most stable gene in set A, B, C and D (Table 3). *Vps26* and *Ras* were alternating at the second and third position of the most stably expressed genes in the three sets containing fruiting body samples (B, C and D), while *ACTB* and *HH3* were the second and third most stable in set A (mycelia set). The two least stable ICGs differed between set A (*18S* and *GAPDH*), B (*CYP* and *ACTB*), and C and D (*CYP* and *GAPDH*) (Table 3).

#### BestKeeper analysis of ICGs

BestKeeper analyzes each gene’s expression variability by calculating the standard deviation (SD) and coefficient of variance (CV), and then determines the most stably expressed genes by the correlation coefficient to the BestKeeper index (the geometric mean of Ct values of candidate ICGs)^26^. Considering the limitations within the program for the number of genes that can be analyzed simultaneously, seven genes with a SD greater than one (*L-asp*, *β-TUB1*, *β-TUB2*, *GAPDH*, *CYP*, *α-TUB* and *ACTB*) were eliminated from the analysis, because of their high variation (Supplementary Table S3). *MSF1* and *18S* were further excluded due to their low correlation coefficients and high *P* values (>0.01) when compared with the BestKeeper index (Table 4). Of the remaining genes, *SPRYp*, *Ras* and *HH3* were the most stable, showing high Pearson correlation coefficients (R > 0.900) and low *P* values (=0.001) (Table 4). BestKeeper analysis of the correlations between any two of the genes suggested gene pairs with very similar overall expression patterns that would be reliable for normalization. The combination of *SPRYp* and *Ras* exhibited the highest correlation, followed by *SPRYp* paired with *HH3*, *Vps26* and *Ras*, and then *SPRYp* and *UBQ* (Supplementary Table S4).

The three statistical programs each indicated *SPRYp* as the most stable gene in all four sample sets. Variations between set A (different strains) with *ACTB* and *HH3*, and sample sets B, C and D with *Ras* and *Vps26* as second or third most stable expressed gene were also supported by all three programs. Correspondingly, the most stable gene pairs were *SPRYp* and *ACTB* in set A (all three programs), and *SPRYp*/*Ras* or *SPRYp*/*Vps26* in set B, C and D (Table 5).

#### Evaluation of the expression stability of novel and traditional ICGs in other filamentous fungi

**Figure 3:**
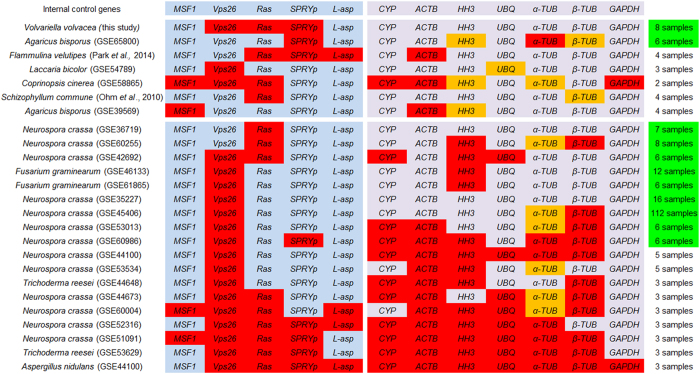
Expression stability of novel and traditional ICGs in other filamentous fungi. Expression stability of thirteen candidate ICGs was examined in twenty-four different RNA-Seq data sets from five Basidiomycetes and four Ascomycetes. Stable and unstable genes were distinguished using an absolute log2-fold change between the maximum and minimum RPKM values ≤ 1 as a threshold. The candidate ICGs with expression variation lower than the threshold are shown in red. Orange indicates the existence of paralogous genes, of which one or more showed expression variation below the threshold. Green indicates the experiments that contained more than five samples and of which the stable genes are expected to be more robust.

To evaluate the stability of the identified ICGs in other fungal species, we analyzed the expression of the respective homologous genes in the available RNA-Seq expression profiles from nine filamentous fungi. Genes of which the RPKM (reads per kilo bases per million reads) values showed less than a twofold change between any of the expression profiles of an experiment (an absolute log2-fold change between the maximum and minimum RPKM values ≤1), were regarded as stable (Fig. 3, marked in red).

Stability of the ICGs in the expression data of Basidiomycetes was irregularly distributed. None of the ICGs was stable in all species, and neither did different ICGs show similar stability in different species. For example, *SPRYp* was stable in *V. volvacea*, *Agaricus bisporus* (GSE65800 35) and *Flammulina velutipes*^36^, but *Vps26* was stable in *V. volvacea*, *Laccaria bicolor* and *Coprinopsis cinerea*. Overall, three of the novel ICGs (*SPRYp*, *Vps26* and *Ras*) and only one traditional ICG (*ACTB*) alternated as the most stable genes in *F. velutipes*, *L. bicolor*, *C. cinerea* and *Schizophyllum commune*^37^ (three to four times non-differential expression over seven experiments, Fig. 3).

For the Ascomycetes, most data was comprised of experiments of *Neurospora crassa* (thirteen out of eighteen). Other expression profiles were obtained from *Fusarium graminearum*, *Trichoderma reesei* and *Aspergillus nidulans*. As expected, more samples in an experimental dataset resulted in fewer ICGs that were not differentially expressed (i.e. not defined as stable). Half of the experiments (nine out of eighteen) included more than five samples (Fig. 3, green), suggesting more robust stability of detected, non-differentially expressed ICGs. Novel ICG *Vps26* was stable in sixteen experiments (out of eighteen), and was found to be stable in seven of the nine experiments with more than five samples. The two traditional ICGs *HH3* and *β-TUB* also showed a robust stability in the experiments with more samples (in five respectively four of the nine experiments, Fig. 3).

## Discussion

The selection of suitable ICGs for RT-qPCR is a prerequisite for reliable gene expression analysis^6^,^38^. Irregular expression was demonstrated for most of the commonly used ICGs that were evaluated in this study (Figs 2 and 3), while others are unsuitable for RT-qPCR normalization due to their high expression level (very low Ct values). *18S* and *28S* are typical examples of genes with undesired expression levels. Ribosomal RNA can constitute up to 80–90% of total cellular RNA^39^, and will under such conditions be expressed at levels that are several orders of magnitude higher than the mean expression of the studied gene(s). Our experiments confirmed that *18S* expression was considerably higher than that of all other ICGs in *V. volvacea* (Supplementary Fig. S1). A careful choice of ICGs that considers the absolute expression level as well as the stability in different samples is therefore important. We started our selection of ICGs based on the DGE data of *V. volvacea*. The three most stable *V. volvacea* genes (*SPRYp*, *Vps26* and *Ras*) were moderately expressed, and showed a roughly tenfold difference in absolute expression (*SPRYp* and *Vps26* compared with *Ras*). Together, they would suit a large selection of genes as a reference. We expect that the increasing availability of RNA-Seq and other expression data will promote the identification of more ICGs with appropriate expression levels, and strongly suggest the inclusion of this measure during selection of ICGs.

With regard to stability, there is little consensus on which kind of algorithm provides the most reliable prediction. We therefore examined the stability of fourteen ICGs with three popular and well cited algorithms, geNorm, BestKeeper and NormFinder, in four different combinations of *V. volvacea* stages. The four datasets represented varying culture conditions and developmental stages of the mushroom forming fungus *V. volvacea*, allowing analysis of ICG expression and their stability in samples with strongly differing gene expression patterns. All three algorithms predicted similar least stable and most stable genes within the same datasets, and as such were consistent (Table 5). Variations in stability rankings generated by the three programs was mainly restricted to genes with intermediate stability rankings, i.e. genes ranked in between the ones that were indicated to be most stable and those that were indicated to be least stably expressed. Likely, differences in the statistical principles of the three programs resulted in small differences in the respective predicted expression stability of genes with closely similar variation. This indicates the importance of selecting multiple ICGs, so that least stable, intermediately stable, and most stable genes can be distinguished. It also stresses the importance of comparing the predicted stabilities that result from different algorithms.

The different composition of the four *V. volvacea* sample sets (A–D) had a large effect on the stability ranking of the analyzed ICGs (Table 5). While the three most stably expressed genes were *SPRYp*, *ACTB* and *HH3* for set A (different strains, Fig. 2), *SPRYp*, *Ras* and *Vps26* where most stable in the remaining three sets containing mushroom tissue, or mycelium and mushroom tissue (Fig. 2b,c,d). Thus, two of the three genes that were indicated as most stable in set A were different from those that were indicated as most stable in set B–D, by all three algorithms. This means that ICGs that have been confirmed to be stable in a certain experimental condition for a species (e.g. mycelium samples), won’t necessarily be stable under different conditions in the same species (e.g. mushroom tissue). Moreover, changes in stability ranking occurred in each newly combined set, including between set C and D that were relatively large and contained mainly the same samples. The suitability of an ICG for a given species is therefore highly dependent on the particular combination of experiments that are analyzed together. This signifies the importance of validating ICGs under the intended experimental conditions.

As expected, the number of ICGs that were non-differentially expressed within the gene expression profiles of the nine filamentous fungi, decreased when more samples were included in an experiment; more expression profiles mean a higher chance of including a condition that affects a particular ICG. Eventually, none of the ICGs was stably expressed in all datasets of the nine different species, or in the datasets of a larger subgroup of species (Ascomycetes or Basidiomycetes). This means that ICGs that are stable even in several species can’t be simply applied in other fungal species. Nonetheless, the three novel ICGs *SPRYp*, *Ras* and *Vps26* showed more robust stability in multiple Basidiomycetous datasets than the traditionally used ICGs, while the novel ICG *Vps26* showed by far the largest stability in the Ascomycetes (followed by the more commonly used *HH3* and *β-TUB* genes). This indicates that the traditionally used ICGs are often not the best choice for normalizing of RT-qPCR data in filamentous fungi, and that reconsideration of ICGs can be rewarding. It might be that the traditionally used ICGs performed better in the analyzed Ascomycete datasets because of their original selection in those species. Similarly, this might explain why the new ICGs that were based on *V. volvacea* data, mostly performed better within Basidiomycetes. Taken together, the identification of a first set of robust ICGs in this study makes a significant contribution to the selection of better ICGs for the normalization of RT-qPCR in filamentous fungi. With the increasing amount of datasets, it should be possible to define generally stable genes with suitable expression levels for most species over time. A set of well-known and tested ICGs can then be selected and quickly examined for any experiment.

We like to conclude with the following recommendations to help to select appropriate ICGs. **1**) It would be preferable to use already existing expression profile data of a species to select ICGs. Alternatively, ICGs could be selected from datasets of closely similar species, especially if these have been used in comparable experiments as the planned research. The benefit is that the absolute expression can be estimated, as well as the rough stability (non-differential expression). If no suitable candidates are available or known, one could start with testing *SPRYp*, *Ras*, *Vps26* and *ACTB* for Basidiomycetes, or *Vps26*, *HH3* or *β-TUB* for Ascomycetes. Moreover, minor differences in absolute expression between an ICG and a target gene (the similar order of magnitude) should be relatively good. The mean TPM or RPKM values of identified ICGs in several filamentous fungi were indicated in Supplementary Table S5. **2**) If possible, genes of known functions should be selected. The prediction of strong fluctuations of expression under the intended experimental conditions based on the gene’s function, could be used to quickly exclude ICG candidates from further testing. For example, as one of the two non-muscle cytoskeletal actins, *ACTB* is involved in cell motility, cell structure and cell integrity^40^. It is therefore likely to be differentially expressed during conditions like starvation or stimulation by growth factors. Testing the suitability of *ACTB* with RT-qPCR for these conditions would be unnecessary. **3**) When selecting ICGs it is advisable to select multiple ICGs, and test them with several algorithms. Truly stable genes will be indicated as such by all programs, while moderately or unstable genes will highly fluctuate in ranking. With only one ICG candidate this will be hard to determine. Testing more than one ICG has the additional benefit that a suitable pair of ICGs can be determined, which improves the reliability of the RT-qPCR normalization. **4**) Species should, whenever possible, be carefully examined for the presence of paralogous genes or other sequences with enough similarity to an ICG to enable a-specific binding of the primers for RT-qPCR^41^. Paralogous genes (indicated in orange in Fig. 3), can show strongly differing expression levels. Also for this reason the selection of ICGs with a known function could be helpful, as the existence of paralogous genes is more likely to be known. **5**) Finally, we like to stress the importance of actually testing the selected ICGs in the conditions of the experiment. Regardless of the selection process, only this will eventually demonstrate if an ICG is suitable.

## Methods

### Organisms, growth conditions and sample harvest

*V. volvacea* homokaryons PYd15 (ACCC52631) and PYd21 (ACCC52632), and heterokaryon H1521 (ACCC52633, a hybrid obtained by mating strains PYd21 and PYd15), were obtained from the Agricultural Culture Collection of China, and maintained, with periodic transfers, on potato dextrose agar, at 20 °C. Mycelium samples of the three strains (Supplementary Table S2) were cultivated in potato dextrose medium with shaking at 33 °C and 120 rpm for four days. For fruiting body production, solid cultures of strain H1521 were cultivated on rice straw compost according to the method of Chen *et al*.^42^. Samples of fruiting bodies at five different developmental stages (primordia, button stage, egg stage, elongation stage and maturation stage, Supplementary Table S2) were harvested according to the method of Tao *et al*.^43^. The whole fruiting body was harvested followed by chopping and mixing; and then the sample in each stage was prepared using a mixture of multiple fruiting bodies. All samples were immediately frozen in liquid nitrogen before RNA extraction. Three independent biological replicates were performed, and all samples in each biological replicate were harvested from a new production batch.

### Screening for traditional and novel ICGs in *V. volvacea*

Nine housekeeping genes, commonly used as ICGs in other studies, were identified in the genome of *V. volvacea* PYd21 (ACCC52632) and confirmed by cross BLAST searches in NCBI. Novel ICG candidates were selected from *V. volvacea* based on DGE profiles of the eight samples (three mycelium samples: PYd21, PYd15, H1521, and five mushroom developmental stages). Genes that were differentially expressed (false discovery rate ≤ 0.001 and an absolute log2-fold change ≥ 1) between any two sets of the eight examined expression profiles, had ten or fewer tag counts in a sample, or were predicted as hypothetical genes or genes of unknown function, were discarded. The remaining genes were analyzed by BLAST analysis in NCBI. The top five candidates, with different orders of magnitude of expression levels, and with the highest similarity with other fungi, were selected for further evaluation.

### RNA isolation, quality control, cDNA synthesis, and design of RT-qPCR primers

Total RNA was isolated using an RNAprep Pure Plant Kit (Tiangen, Beijing, China), according to the manufacturer’s protocol. Extracted RNA was quantified using NanoND-1000 spectrophotometer (NanoDrop Technologies, Wilmington, DE, USA) and analyzed for integrity using agarose gel electrophoresis. Only RNA samples with A260/A280 ratios between 1.9 and 2.1 and A260/A230 ratios greater than 2.0 as well as the brighter *28S* band (compared with *18S* band in agarose gel electrophoresis) were used for cDNA synthesis. And 1 μg of total RNA were used to reverse transcribe into 20 μl cDNA using random primers and an oligo dT primer using a PrimeScript RT reagent Kit with gDNA Eraser (Takara, Shiga, Japan), according to the manufacturer’s protocol. Primer pairs (Table 2) were designed to amplify across introns using DNAMAN 6.0, based on the gene structures according to the genome sequence and transcriptome raw reads obtained by Illumina sequencing using ZOOM software^44^.

### RT-qPCR amplification

RT-qPCR was performed using a CFX96 Real-Time PCR Detection System (Bio-Rad, Hercules, CA, USA) with SsoAdvanced SYBR Green Supermix (Bio-Rad). Amplifications were performed for each sample with three technical replicates, including no-template controls. All amplifications included denaturation for 10 s at 95 °C, followed by 40 cycles of 5 s at 95 °C and 30 s at primer-specific annealing temperatures. The specificity of each RT-qPCR reaction was tested using a melting curve (gradient from 60 to 95 °C), and the presence of a single PCR product was verified by 2% agarose gel electrophoresis. Standard curves of a minimum of five points and sixfold dilution series from pooled cDNA were used to calculate the PCR efficiency.

### Data processing and statistical analysis

The RT-qPCR Ct value of each gene was obtained by calculating the arithmetic mean from three technological and three biological replicates. The arithmetic mean Ct values were converted to relative copy numbers using the standard curve method^3^ for geNorm and NormFinder analyses. Amplification efficiencies (E) and correlation coefficients (R^2^ values) were obtained from standard curves. Algorithms geNorm version 3.5, NormFinder version 0.953, and BestKeeper version 1, were used to evaluate the expression stabilities of the candidate ICGs.

To investigate the stability of ICGs under different sample compositions, four experimental data sets were generated, comprising varying combinations of the eight *V. volvacea* samples (Supplementary Table S2). Set A included the mycelium samples of the three different *V. volvacea* strains PYd15, PYd21 and H1521, for comparison between strains. Set B included the samples from the five different developmental stages of the fruiting body (all from strain H1521), to validate ICGs whose expression levels were not influenced by developmental differences. Set C was similar to set B but included one additional sample, the H1521 mycelium, which combined samples of different substrates and different environmental conditions. Set D included all samples, combining different strains, different growth conditions and different developmental stages.

### *In-silico* analysis of RNA-Seq data

To identify the homologues of the thirteen selected ICGs (except for *18S*), a standard Local BLAST (blastp) using the amino-acid sequences from *V. volvacea* was performed against protein databases of the other filamentous fungi (from the JGI database). The detailed lists of genes or proteins ID for each ICG in every fungus are available in the Supplementary Table S5. For each ICG, the RPKM values were obtained from the RNA-Seq data in each experiment, and analyzed for the expression stability. A total of twenty-two RNA-Seq data sets from the seven filamentous fungi (three Basidiomycetes: *A. bisporus*, *L. bicolor*, *C. cinerea*, and four Ascomycetes: *N. crassa*, *F. graminearum*, *T. reesei*, *A. nidulans*) were publicly available at the NCBI gene expression omnibus (GEO) database, and the accession numbers are indicated in Fig. 3 and Supplementary Table S5. In addition, the expression data from *F. velutipes* and *S. commune* were obtained from Park *et al*.^36^ and Ohm *et al*.^37^, respectively. Expression variation of the thirteen ICGs was assigned using a threshold of an absolute log2-fold change between the maximum and minimum RPKM values ≤ 1.

## Additional Information

**Accession codes:** The draft genome of *V. volvacea* PYd21 (ACCC52632) is available at GenBank accession no. PRJNA171553. Digital gene expression raw data of homokaryon PYd21 and PYd15, and heterokaryon H1521 are deposited at gene expression omnibus (GEO) database: GSM1055162, GSM1055163, GSM1055164, http://www.ncbi.nlm.nih.gov/geo/query/acc.cgi?token=xtoxncacgwqewnc&acc=GSE43019. The raw digital gene expression data of five developmental stages of *V. volvacea* are deposited at GEO database:GSM1060232-GSM1060236, http://www.ncbi.nlm.nih.gov/geo/query/acc.cgi?token=pxcbpqmmwqeuopo&acc=GSE43297. The genes identified in this study have been deposited in GenBank (Table 2).

**How to cite this article**: Tao, Y. *et al*. Identification of novel and robust internal control genes from *Volvariella volvacea* that are suitable for RT-qPCR in filamentous fungi. *Sci. Rep.* **6**, 29236; doi: 10.1038/srep29236 (2016).

## Supplementary Material

Supplementary Information

## Figures and Tables

**Table 1:** The average expression levels (TPM) in *V. volvacea* and the predicted function of the five newly selected ICGs.

Gene symbol	Mean TPM	Gene name	Pfam annotation	KOG annotation	Function
*L-asp*	6.30	L-asparaginase	Asparaginase (PF00710)	Asparaginase; Amino acid transport and metabolism	Catalyze the hydrolysis of asparagine
*Ras*	7.88	Ras protein	Ras family (PF00071)	Ras-related small GTPase; General function prediction only	Regulate signaling networks involved in actin organization, cell proliferation, differentiation, vesicular trafficking
*SPRYp*	61.76	Endosomal SPRY domain protein	SPRY domain (PF00622)	SPRY domain-containing proteins; General function prediction only	Be involved in cargo recognition and nutrient transport as a component of the endosome-vacuole trafficking pathway
*Vps26*	89.46	Vacuolar protein sorting-associated protein 26	Vacuolar protein sorting-associated protein 26 (PF03643)	Membrane coat complex Retromer, subunit VPS26; Intracellular trafficking, secretion, and vesicular transport	Participate in protein trafficking to the vacuole and mediate cargo selection
*MSF1*	156.28	MSF1 domain containing protein	PRELI-like family (PF04707)	Member of the intra-mitochondrial sorting protein family; Intracellular trafficking, secretion, and vesicular transport	Be involved in intra-mitochondrial protein sorting; maintain mitochondrial electron transport chain and respiratory competency

**Table 2:** Descriptions of fourteen candidate ICGs in *V. volvacea* and parameters derived from RT-qPCR analysis.

Gene symbol	Gene name	Accession number	Primer sequences (5′–3′)	Product size (bp)	PCR efficiency (100%)^a^	Annealing temperature (°C)
*18S*	18S ribosomal RNA	KF471312	F: TCTTGTGAAACTCTGTCGTGCTGGGR: TTGCCCACACCCCAAAGCTAATTCG	186	1.87	66
*ACTB*	β-actin	KF528321	F: AGCTCTCTTCCAACCTGCCTTCTTGR: GCGACAATCTTAACCTTCATGCTTGC	216	1.98	62
*CYP*	Cyclophilin	KF528322	F: GATCCCAATTCTTCGTCACCACTGR: ACCACTTGCAGATCCAAAGCCCTCG	125	1.97	63
*GAPDH*	Glyceraldehyde 3-phosphate dehydrogenase	KF528323	F: ATTGGCGTGGTGGTCGTAGR: ACGGAAACATCAAGGGTAGGG	142	1.95	60
*HH3*	Histone H3	KF528324	F: GTGAAATCAGACGTTACCAGAAGTR: TGGGTTGGATAGTGACACGCTTAG	219	2.03	59
*α-TUB*	α-tubulin	KF528325	F: ACTTGGTTCCTTTCCCTCGTATTCACR: GCCATGTATTTGCCTTCCCTAG	169	2.02	61
*β-TUB1*	β-tubulin 1	KF528326	F: GATTCCCAACAACGTCCTCACCR: TCAGCTTCCGTGAACTCCATCTC	204	1.90	61
*β-TUB2*	β-tubulin 2	KF528327	F: GAACTCAAGAGACATTCGCTGCCR: TTCACCATCCTCCACAGACGCTTC	164	1.94	63
*UBQ*	Ubiquitin	KF528328	F: GGAAAGACGATCACCTTGGAGR: AGGCGAAGAACAAGATGAAGAGTG	191	1.95	60
*L-asp*	L-asparaginase	KF528333	F: TTCGTGTCGCTCTCTCTCCAGR: GTATTGACTGTTTGTGAGGCTTGT	141	2.00	59
*Ras*	Ras protein	KF528332	F: ACATACGAGAGAGAAGTTTCCAAGR: TCTCCTCCTTCTGTGGCTTGAC	181	2.13	59
*SPRYp*	Endosomal SPRY domain protein	KF528330	F: CTCCTCCTCCAATAACCCCCATCR: TCAGAGTCTATTCCTTGCCTATCCG	181	2.03	62
*Vps26*	Vacuolar protein sorting associated protein 26	KF528331	F: CGATTCGACTGTTCCTTGGTGGR: TGCTTGAAATATCGGCGGTTCTC	127	1.99	61
*MSF1*	MSF1 domain containing protein	KF528329	F: CAGATGGACTAGAGAAGTGGTTAGR: ATCTCTTATGAAGGTGCGTGTG	136	1.94	59

^a^2 represents PCR amplification efficiency is 100%.

**Table 3:** Stability analysis of fourteen candidate ICGs using NormFinder analysis.

Sample sets	Different strains set (A)		Different fruiting body developmental stages set (B)		Different growth stages set (C)		Total samples set (D)	
Rank	Gene	Stabilityvalue	Gene	Stabilityvalue	Gene	Stabilityvalue	Gene	Stabilityvalue
1	*SPRYp*	0.049	*SPRYp*	0.078	*SPRYp*	0.071	*SPRYp*	0.094
2	*ACTB*	0.057	*Vps26*	0.131	*Ras*	0.208	*Vps26*	0.204
3	*HH3*	0.074	*Ras*	0.209	*Vps26*	0.219	*Ras*	0.209
4	*L-asp*	0.154	*L-asp*	0.240	*UBQ*	0.287	*UBQ*	0.279
5	*Ras*	0.175	*β-TUB2*	0.241	*HH3*	0.341	*HH3*	0.284
6	*β-TUB1*	0.178	*18S*	0.320	*β-TUB2*	0.363	*β-TUB2*	0.532
7	*UBQ*	0.273	*UBQ*	0.346	*L-asp*	0.517	*L-asp*	0.554
8	*β-TUB2*	0.316	*HH3*	0.393	*18S*	0.518	*MSF1*	0.636
9	*α-TUB*	0.335	*GAPDH*	0.505	*MSF1*	0.653	*ACTB*	0.749
10	*MSF1*	0.424	*β-TUB1*	0.582	*β-TUB1*	0.661	*β-TUB1*	0.780
11	*CYP*	0.442	*MSF1*	0.670	*α-TUB*	0.794	*18S*	0.849
12	*Vps26*	0.444	*α-TUB*	0.786	*ACTB*	0.811	*α-TUB*	0.886
13	*GAPDH*	0.458	*ACTB*	0.812	*GAPDH*	1.011	*GAPDH*	1.231
14	*18S*	0.587	*CYP*	1.077	*CYP*	1.192	*CYP*	1.300

**Table 4:** Stability analysis of seven candidate ICGs using BestKeeper analysis.

	*Ras*	*SPRYp*	*Vps26*	*MSF1*	*18S*	*HH3*	*UBQ*	BestKeeper index
N	8	8	8	8	8	8	8	8
Std. dev [±Ct]	0.70	0.77	0.63	0.82	0.83	0.91	0.67	0.54
CV [%Ct]	3.61	3.38	2.86	3.97	7.16	4.98	4.19	2.95
Coeff. of corr. [R]	0.918	0.937	0.841	0.636	0.124	0.917	0.892	–
*P* value	0.001	0.001	0.009	0.090	0.767	0.001	0.003	–

N: number of samples; Std dev [±Ct]: standard deviation of the Ct; CV (%Ct): the coefficient of variance expressed as a percentage of the Ct level. The correlation between each candidate gene and the BestKeeper index was calculated using the Pearson correlation coefficient [R] and the *P* value.

**Table 5:** Comparison of the results of geNorm, BestKeeper and NormFinder analysis.

Sample sets	The top five most stable genes	Most stable combination of two genes	The two least stable genes
Different strains set (A)	*SPRYp*, *ACTB*, *HH3*, *Ras*, β-TUB1*, (*L-asp*)**	*SPRYp *+ *ACTB*	*18S*, *GAPDH*
Different fruiting body developmental stages set (B)	*SPRYp*, *Ras*, *Vps26*, *L-asp*, *β-TUB2*	(*SPRYp *+ *Ras*)*(*SPRYp *+ *Vps26*)**	*CYP*, *ACTB*
Different growth stages set (C)	*SPRYp*, *Ras*, *Vps26*, L-asp*, β-TUB2* (*UBQ*, *HH3*)**	*SPRYp *+ *Ras*	*CYP*, *GAPDH*
Total samples set (D)	*SPRYp*, *Ras*, *Vps26*, *HH3*, β-TUB2* (*UBQ*)**	(*SPRYp *+ *Ras*)*^,#^(*SPRYp *+ *Vps26*)**	*CYP*, *GAPDH*

*^,#^ and ** indicate that ICG was estimated by geNorm, BestKeeper and NormFinder, respectively.
