# Supplementary material for: Identification of novel and robust internal control genes from Volvariella volvacea that are suitable for RT-qPCR in filamentous fungi
Source: Sci Rep. 2016 Jul 12;6:29236. doi: 10.1038/srep29236 (PMC4941408; doi:10.1038/srep29236)
Supplement: Supplementary Information [file srep29236-s1.pdf]

Supplementary Information

Identification of novel and robust internal control genes from *Volvariella volvacea* that are suitable for RT-qPCR in filamentous fungi

Yongxin Tao[1,2], Arend Frans van Peer[2], Qianhui Huang[1,2], Yanping Shao[1,2], Lei Zhang[2], Bin Xie[2], Yuji Jiang[2,3], Jian Zhu[1,2], Baogui Xie[2,*]

[1] College of Horticulture, Fujian Agriculture and Forestry University, Fuzhou, 350002, Fujian, China

[2] Mycological Research Center, College of Life Sciences, Fujian Agriculture and Forestry University, Fuzhou, 350002, Fujian, China

[3] College of Food Science, Fujian Agriculture and Forestry University, Fuzhou, 350002, Fujian, China

* Corresponding author: mrcfafu@163.com (B. X)

To Corresponding author: (Baogui Xie)

Mycological Research Center, College of Life Sciences, Fujian Agriculture and Forestry University, Fuzhou 350002, China

Tel.: +86 591 83789277; fax: +86 591 83789277

E-mail address: mrcfafu@163.com

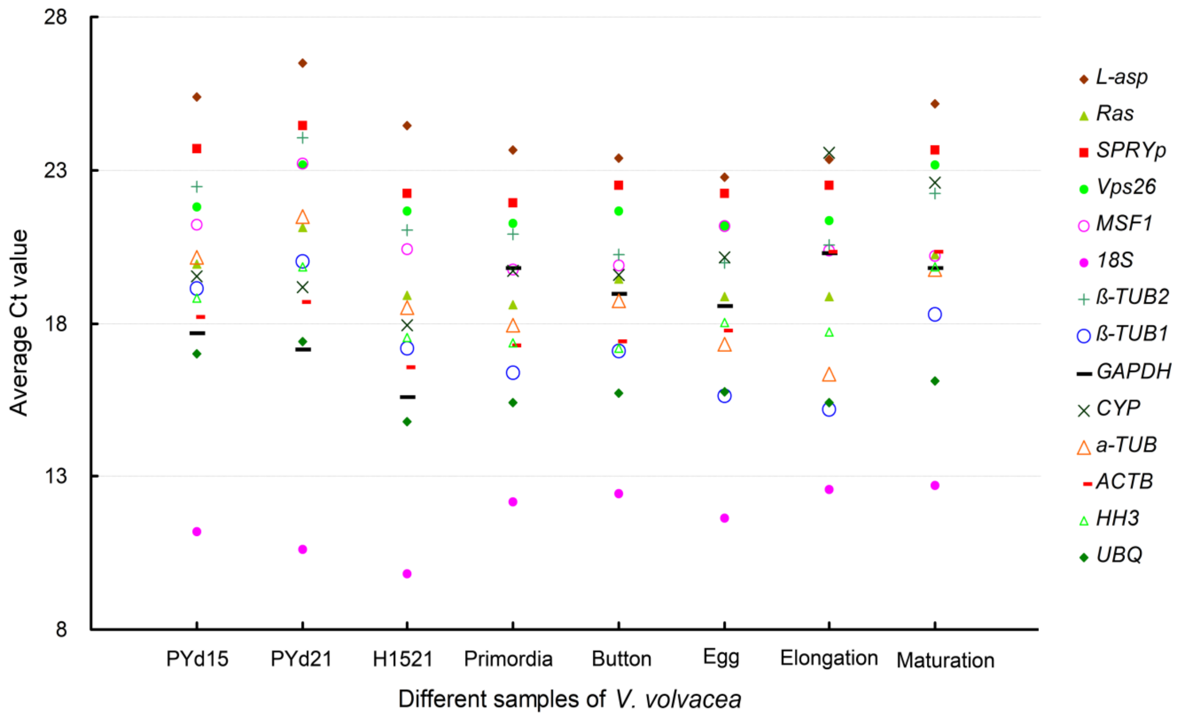

Figure S1. RT-qPCR Ct values of fourteen ICGs in eight samples of *V. volvacea*

Mean Ct values of three biological replicates and three technical replicates are shown on the Y-axis. Different samples are shown on the X-axis: PYd15, homokaryon PYd15 mycelium; PYd21, homokaryon PYd21 mycelium; H1521, heterokaryon H1521 mycelium; primordia, button stage, egg stage, elongation stage and maturation stage are five developmental stages of fruiting bodies of *V. volvacea* strain H1521.

Table S1. The expression levels (TPM values, from digital gene expression profiles) of eight traditional housekeeping genes among eight expression profiles of *Volvariella volvacea*

Gene	PYd15 (TPM)	PYd21 (TPM)	H1521 (TPM)	Primordia (TPM)	Button stage (TPM)	Egg stage (TPM)	Elongation stage (TPM)	Maturation stage (TPM)	LOG_2 (MAX/MIN)
ACTB	2016.04	1220.49	2285.6	1549.14	707.55	750.85	173.96	505.87	3.72
CYP	1562.26	1066.39	1533.38	694.61	235.2	199.88	20.26	156.84	6.27
GAPDH	3923.63	6511.45	4756.06	541.06	389.32	341.89	155.88	942.53	5.38
HH3	111.58	410.69	88.22	125.63	68.06	227.15	90.75	44.91	3.19
α-TUB	9.91	3.53	6.66	0.71	4.28	19.4	17.92	5.53	4.77
β-TUB1	12.69	7.56	8.94	3.89	24.23	34.96	41.19	4.52	3.40
β-TUB2	78.21	69.74	131.54	78.28	82.14	257.42	49.9	84.45	2.37
UBQ	38.06	129.56	29.11	61.32	47.93	52.69	50.73	59.48	2.15

Table S2. Eight samples of *Volvariella volvacea* involved in this study and the four different combinations of sample sets

No.	Materials	Notes
1	PYd15 mycelium	Homokaryon
2	PYd21 mycelium	Homokaryon
3	H1521 mycelium	Heterokaryon
4	Primordia	Fruiting body of strain H1521
5	Button stage	Fruiting body of strain H1521
6	Egg stage	Fruiting body of strain H1521
7	Elongation stage	Fruiting body of strain H1521
8	Maturation stage	Fruiting body of strain H1521
The four different combinations of sample sets:		
A	Different strains set	Samples of 1, 2 and 3
B	Different fruiting body developmental stages set	Samples of 4, 5, 6, 7 and 8
C	Different growth stages set	Samples of 3, 4, 5, 6, 7 and 8
D	Total samples set	Samples of 1, 2, 3, 4, 5, 6, 7 and 8

Table S3. Standard deviation of Ct values of fourteen candidate internal control genes using BestKeeper program

	L-asp	*Ras*	*SPRYp*	*Vps26*	*MSF1*	*18S*	*β-TUB2*	*β-TUB1*	*GAPDH*	*CYP*	*α-TUB*	*ACTB*	*HH3*	*UBQ*
N	8	8	8	8	8	8	8	8	8	8	8	8	8	8
Geo Mean [Ct]	24.31	19.48	22.88	21.89	20.75	11.61	21.40	17.30	18.42	20.21	18.72	18.27	18.26	15.93
Ar Mean [Ct]	24.34	19.50	22.90	21.90	20.78	11.65	21.44	17.37	18.48	20.28	18.78	18.31	18.29	15.95
Min [Ct]	22.76	18.61	21.93	21.18	19.73	9.83	19.98	15.20	15.59	17.95	16.34	16.56	17.20	14.79
Max [Ct]	26.49	21.13	24.44	23.16	23.22	12.72	24.06	20.01	20.30	23.57	21.50	20.33	19.85	17.41
Std dev [±Ct]	1.04	0.70	0.77	0.63	0.82	0.83	1.11	1.34	1.26	1.40	1.27	1.10	0.91	0.67
CV [% Ct]	4.27	3.61	3.38	2.86	3.97	7.16	5.19	7.70	6.83	6.91	6.77	6.00	4.98	4.19

N: number of samples; Geo Mean: geometric mean of Ct values; Ar Mean: arithmetic mean of Ct values; Std dev [±Ct]: the standard deviation of the Ct; CV (%Ct): the coefficient of variance expressed as a percentage of the Ct level. The seven genes (*L-asp*, *β-TUB1*, *β-TUB2*, *GAPDH*, *CYP*, *α-TUB* and *ACTB*) with highest standard deviation values (SD>1) are highlighted with red and removed in the first step (SD higher than 1 means high variation in expression).

Table S4. Correlation analysis of optimal internal control gene pairs using BestKeeper analysis

vs.	*Ras*	*SPRYp*	*Vps26*	*MSF1*	*18S*	*HH3*	*UBQ*
SPRYp	0.971	—	—	—	—	—	—
P value	0.001	—	—	—	—	—	—
Vps26	0.913	0.863	—	—	—	—	—
P value	0.002	0.006	—	—	—	—	—
MSF1	0.714	0.725	0.506	—	—	—	—
P value	0.047	0.042	0.200	—	—	—	—
18S	-0.189	-0.172	-0.094	-0.541	—	—	—
P value	0.655	0.682	0.826	0.165	—	—	—
HH3	0.877	0.914	0.892	0.644	-0.093	—	—
P value	0.004	0.002	0.003	0.085	0.826	—	—
UBQ	0.862	0.901	0.628	0.768	-0.109	0.778	—
P value	0.006	0.002	0.095	0.026	0.796	0.023	—

Table S5. The average absolute expression levels (TPM or RPKM values) of homologous genes of thirteen ICGs (except *18S*)

Internal control genes	Gene or protein ID	Mean TPM or RPKM
Volvariella volvacea	Gene ID	This study
L-asp	GME5360_g	6.30
Ras	GME11562_g	7.88
SPRYp	GME11387_g	61.76
Vps26	GME1490_g	89.46
MSF1	GME11630_g	156.28
β-TUB1	GME2992_g	17.25
β-TUB2	GME1291_g	103.96
GAPDH	GME5082_g	2195.23
CYP	GME7263_g	683.60
α-TUB	GME8406_g	8.49
ACTB	GME9359_g	1151.19
HH3	GME9377_g	145.87
UBQ	GME11487_g	58.61

Flammulina velutipes	ORF ID	Park *et al*., 2014
L-asp	ctg11-2_AA_00476	3.62
Ras	ctg03_AA_00737	69.67
SPRYp	ctg03_AA_00882	29.53
Vps26	ctg11-1_AA_01092	11.83
MSF1	ctg03_AA_00662	168.11
β-TUB-1	ctg03_AA_00282	76.87
β-TUB-2	ctg01_AA_00278	536.67
GAPDH	ctg06_AA_00270	632.18
CYP	ctg05_AA_01318	1617.44
α-TUB-1	ctg05_AA_00452	134.12
α-TUB-2	ctg11-2_AA_00851	38.10
ACTB	ctg11-1_AA_00516	534.92
HH3-1	ctg13_AA_00215	254.72
HH3-2	ctg13_AA_00224	192.81
UBQ	ctg03_AA_00799	371.22

Agaricus bisporus	Protein ID	GSE65800	GSE39569
L-asp	63743	37.50	4427.50
Ras	133440	412.98	55015.00
SPRYp	60883	67.09	72.50
Vps26	194951	37.76	12820.50
MSF1	189859	217.21	2643.00
β-TUB-1	195658	850.36	27686.25
β-TUB-2	183821	208.21	8254.00
β-TUB-3	187460	7.94	821.00
GAPDH	138631	1388.87	81562.75
CYP	138080	622.31	82610.50
α-TUB	135559	694.00	41241.75
ACTB	192120	899.53	51573.00
HH3-1	136889	125.63	5537.75
HH3-2	139997	772.18	18438.25
HH3-3	136959	189.30	28760.25
UBQ	133168	4393.59	101109.75

Coprinopsis cinerea	Gene ID	GSE58865
L-asp	CC1G_01149	658.59
Ras	CC1G_04430	1116.54
SPRYp	CC1G_06751	1849.61
Vps26	CC1G_09827	88.86
MSF1	CC1G_01701	532.76
β-TUB-1	CC1G_04743	775.51
β-TUB-2	CC1G_06184	67.91
GAPDH	CC1G_09116	4968.23
CYP	CC1G_09572	3299.42
α-TUB-1	CC1G_01375	190.76
α-TUB-2	CC1G_00146	611.51
ACTB	CC1G_08232	3460.98
HH3-1	CC1G_04396	9.67
HH3-2	CC1G_08799	4852.32
HH3-3	CC1G_05766	782.95
UBQ-1	CC1G_03676	115378.52
UBQ-2	CC1G_11833	4054.82

Laccaria bicolor	Protein ID	GSE54789
L-asp	244034	4.64
Ras-1	291884	48.17
Ras-2	254501	1.49
Ras-3	256615	1.11
SPRYp	228899	0.96
Vps26	173065	70.81
MSF1	192412	254.62
β-TUB-1	294746	178.47
β-TUB-2	191405	32.93
GAPDH-1	295504	132.60
GAPDH-2	318873	335.16
CYP-1	294609	577.41
CYP-2	333157	3.86
α-TUB-1	192523	429.83
α-TUB-2	192524	69.92
ACTB-1	192701	532.52
ACTB-2	303826	9.77
HH3-1	170733	88.36
HH3-2	302849	52.13
HH3-3	294559	226.81
HH3-4	191988	90.97
UBQ-1	186716	1781.31
UBQ-2	192623	2716.81

Schizophyllum commune	Protein ID	Ohm *et al*., 2010
L-asp	66334	5.20
Ras	45883	74.07
SPRYp	64633	13.96
Vps26	77215	68.11
MSF1	73448	413.34
β-TUB-1	77035	1113.84
β-TUB-2	108932	164.41
GAPDH	78936	587.51
CYP	78835	1953.41
α-TUB-1	9815	286.28
α-TUB-2	72168	210.80
ACTB	83632	49.38
HH3-1	47315	28.07
HH3-2	67329	0.00
HH3-3	79839	0.00
HH3-4	27888	118.09
HH3-5	84991	97.27
UBQ-1	85500	2051.99
UBQ-2	64979	1475.51

Aspergillus nidulans	Gene_Locus	GSE44100
L-asp	AN8169	40.73
Ras	AN5832	92.27
SPRYp	AN0831	81.20
Vps26	AN3642	41.33
MSF1	AN4295	259.37
β-TUB-1	AN1182	287.63
β-TUB-2	AN6838	131.54
GAPDH	AN8041	881.73
CYP-1	AN8605	3355.55
CYP-2	AN3814	392.60
α-TUB-1	AN0316	259.28
α-TUB-2	AN7570	220.80
ACTB	AN6542	663.66
HH3	AN0733	2515.32
UBQ	AN2000	2000.65

Fusarium graminearum	Gene_Locus	GSE46133	GSE61865
L-asp	FGSG_07410	27.12	8.74
Ras	FGSG_10114	185.16	56.55
SPRYp	FGSG_01852	48.67	72.03
Vps26	FGSG_01155	65.55	66.35
MSF1	FGSG_10319	171.85	636.59
β-TUB-1	FGSG_09530	319.05	230.23
β-TUB-2	FGSG_06611	479.90	179.09
GAPDH	FGSG_06257	#N/A	2048.36
CYP	FGSG_00777	2278.81	806.81
α-TUB-1	FGSG_00639	502.80	239.65
α-TUB-2	FGSG_00397	635.39	208.75
ACTB	FGSG_07335	1395.45	849.19
HH3	FGSG_04290	3462.17	4262.53
UBQ	FGSG_08768	1581.34	1535.84

Trichoderma reesei	Gene ID	GSE44648	GSE53629
L-asp	e_gw1.28.60.1	0.28	408.06
Ras	fgenesh5_pg.C_scaffold_23000098	5.05	1293.07
SPRYp	estExt_Genewise1.C_180178	8.92	3869.05
Vps26	e_gw1.12.211.1	2.20	1464.05
MSF1	estExt_GeneWisePlus.C_270244	20.98	3792.30
β-TUB-1	estExt_fgenesh5_pg.C_160102	24.57	3357.87
β-TUB-2	estExt_fgenesh1_pm.C_40092	20.78	4049.87
GAPDH	estExt_fgenesh5_pg.C_10410	166.09	43090.21
CYP	estExt_fgenesh5_pg.C_100037	63.22	6086.60
α-TUB	estExt_fgenesh5_pg.C_40423	9.10	5274.46
ACTB	estExt_Genewise1.C_11839	80.60	19940.54
HH3	estExt_fgenesh5_pg.C_390017	49.34	17729.02
UBQ	estExt_fgenesh5_pg.C_140156	130.28	45349.08

Neurospora crassa	Protein ID	GSE36719	GSE42692	GSE35227	GSE45406	GSE53013	GSE60986	GSE44100	GSE53534	GSE44673	GSE60004	GSE52316	GSE51091
L-asp	NCU03768	72.89	46.50	27.66	59.99	35.67	11.03	10.56	20.47	7.61	2.35	4.10	21.34
Ras	NCU03616	279.27	341.45	358.71	99.95	47.22	40.57	47.63	15.47	62.38	53.84	39.07	72.48
SPRYp	NCU03678	418.47	318.04	358.76	51.40	18.07	19.36	61.92	22.01	68.41	57.18	85.49	36.61
Vps26	NCU02743	332.95	354.06	459.59	101.42	41.42	34.80	55.00	16.06	64.29	33.94	43.78	39.99
MSF1	NCU04307	321.26	238.71	140.23	100.92	19.24	18.59	19.80	7.81	42.22	13.45	83.47	39.85
β-TUB-1	NCU04054	4244.25	4298.39	4393.86	1032.81	781.86	679.69	572.29	357.00	603.88	244.14	498.89	675.09
β-TUB-2	NCU04054	4244.25	4298.39	4393.86	1032.81	781.86	679.69	572.29	357.00	603.88	244.14	498.89	675.09
GAPDH	NCU01528	39886.06	28916.09	35633.73	3596.83	4027.27	2471.88	4029.20	16553.05	3931.17	1367.84	2725.67	3795.69
CYP	NCU00726	17626.09	14551.15	19694.72	3740.68	3455.26	2060.09	2699.91	2017.39	2639.27	1315.66	2596.72	1782.67
α-TUB-1	NCU09468	2144.33	2157.97	2595.15	524.84	541.01	286.44	330.92	203.35	269.16	127.40	261.33	306.31
α-TUB-2	NCU09132	2721.44	2690.19	2649.47	778.30	435.10	336.48	312.43	174.84	317.88	138.15	229.98	340.93
ACTB	NCU04173	6631.77	5579.17	3537.84	2007.65	1354.89	1174.95	1361.49	591.89	907.73	528.72	1040.19	1068.38
HH3	NCU01635	15822.65	15619.89	17496.40	6499.16	2385.95	2112.48	2459.12	1389.77	3300.80	2239.55	1530.28	2393.35
UBQ	NCU05995	6903.87	4328.64	7529.56	1366.28	488.72	308.99	768.61	305.97	1053.46	372.59	1146.08	423.30
Internal control genes	Gene or protein ID	Mean TPM or RPKM											
